# The profile analysis of circular RNAs in cervical cancer

**DOI:** 10.1097/MD.0000000000027404

**Published:** 2021-10-01

**Authors:** Jinbing Huang, Junying Chen, Qiaoqiao Huang

**Affiliations:** Department of Gynecology, The First Affiliated Hospital of Guangxi Medical University, Nanning, Guangxi Province, China.

**Keywords:** diagnostic markers, endogenous regulation, functional analysis, Gene Expression Omnibus, PCR validation

## Abstract

Cervical cancer (CC) is the third most common cancer among women and has a high mortality rate at the advanced stage. The mechanisms underlying the development and progression of CC are still elusive. Circular RNAs (circRNAs) play an important role in various physiological and pathological processes. The aim of this study was to identify the circRNAs significantly associated with cervical squamous cell carcinoma (CSCC), in order to discover novel diagnostic markers and elucidate their mechanistic basis.

The circRNA expression profiles of CSCC and paired para-cancerous cervical tissues was downloaded from the Gene Expression Omnibus. Bioinformatics analysis were used to screen for the differentially expressed circRNAs (DECRs). The expression levels of hsa_circ_0000745, hsa_circ_0084927, hsa_circ_0002762, hsa_circ_0075341, hsa_circ_0007905, hsa_circ_0031027, hsa_circ_0065898, hsa_circ_0070190, and hsa_circ_0078383 were verified in CC and normal cervical tissues by quantitative real-time PCR.

A total of 197 DECRs were identified between the CSCC and normal tissues, including 87 upregulated and 110 downregulated circRNAs. In addition, 37 miRNAs were predicted for the upregulated circRNAs and 39 for the downregulated circRNAs. Functional analysis showed that the DECRs were associated with positive regulation of substrate adhesion-dependent cell spreading, metabolism, positive regulation of GTPase activity, protein regulation, and intercellular adhesion. The MAPK signaling pathway that plays a significant role in the progression of CC, was also enriched. Consistent with the in-silico analysis, hsa_circ_0000745, hsa_circ_0084927, hsa_circ_0002762, hsa_circ_0007905 were upregulated and hsa_circ_0078383 was downregulated in CC tissues (*P* < .001), whereas hsa_circ_0075341 (*P* < .001) and hsa_circ_0031027 (*P* = .001) showed opposite trends.

We identified novel diagnostic and therapeutic biomarkers of CSCC along with the mechanistic basis.

## 1 Introduction

Cervical cancer (CC) is the third most common cancer among women worldwide, with over 569,800 new cases and 311,400 deaths recorded in 2018 alone.^[1]^ Cervical squamous cell carcinoma (CSCC) accounts for more than 85% of all cases. Although HPV vaccines have shown encouraging results in preventing CC, it remains the fourth most common cause of cancer-related deaths in women.^[1,2]^ While the 5-year survival rate of localized CC is 91.5%, it declines to 16.5% following metastasis.^[3,4]^ The satisfactory prognosis in the early stages of CC can be attributed to the recent advances in conventional treatment strategies.^[5–7]^ However, approximately two-thirds of the CC patients are diagnosed at the advanced stage due to lack of practical diagnostic biomarkers,^[8]^ which makes the standard treatment strategies ineffectual. Therefore, it is essential to screen for new diagnostic/therapeutic biomarkers in order to improve prognosis of CSCC.

Circular RNAs (circRNAs) are a category of noncoding RNAs formed via the head-to-tail splicing of exons, which result in covalently closed continuous loops lacking both the 5′ caps and 3′ polyadenylated tails.^[9,10]^ Recent studies show that circRNAs regulate tumor cell proliferation, survival and metastasis as tumor suppressors or oncogenes.^[11]^ Hansen et al^[12]^ showed that circRNAs, acting as microRNA sponges and regulate the expression of target mRNAs post-transcriptionally. The circRNA BCRC-3 inhibits bladder cancer proliferation via the miR-182-5p/p27 axis,^[13]^ while circABCB10 acts as an oncogene in osteosarcoma by sponging miR-203, and facilitates nonsmall cell lung cancer cell progression and migration through the miR-1252/FOXR2 axis.^[14,15]^ Li et al^[16]^ recently reported that several circRNAs are aberrantly expressed in the early stage of bladder cancer, and are therefore promising biomarkers for early diagnosis. In addition, there is also evidence indicating both oncogenic and suppressive functions of specific circRNAs in CC. For instance, circ0023404 inhibits CC cell proliferation, cell cycle progression, migration, and invasion by targeting the mir-136/TFCP2/YAP pathway,^[17]^ while circRNA8924, promotes the malignant and invasive behavior of CC cells by modulating CBX8 as a competitive endogenous (ce)RNA to miR-518d-5p/519-5p.^[18]^

Taken together, it is essential to screen for the circRNAs associated with CC development and progression, in order to identify potential biomarkers for early diagnosis and targeted therapy. Li et al^[19]^ recently profiled circRNAs using the microarray tool. To this end, we obtained the circRNA expression data of CSCC and healthy tissues from the Gene Expression Omnibus (GEO) database, and identified the differentially expressed circRNAs (DECRs) in CSCC. In addition, we also identified the functions of the relevant circRNAs, and elucidated the circRNA-miRNA network potentially involved in CSCC progression.

## 2 Methods

### 2.1 Identification of DECRs and the target miRNAs

The circRNA expression data of 5 pairs of CSCC and the corresponding para-carcinoma cervical tissues, based on the GPL19978 Agilent-069978 Arraystar Human CircRNA microarray V1 platform (Agilent Technologies Inc., MD), was downloaded from the GEO database (accession no. GSE102686).GEO2R (https://www.ncbi.nlm.nih.gov/geo/geo2r/) of the R limma package was used to screen for the DECRs in CSCC, with log |FC| > 1 and *P* < .05 as the thresholds.^[20,21]^ The target miRNAs of the DECRs were predicted using the online tool CircInteractome (https://circinteractome.nia.nih.gov/).^[22]^ In addition, studies published till January 20, 2019 on CC-related miRNAs were extracted from Pubmed using the key words “cervical cancer” and “miRNA”. Finally, the co-expressing miRNAs of the 2 datasets were used as the target miRNAs of DECRs.

### 2.2 Construction of the circRNA–miRNA–target gene interaction network

The circRNA–miRNA–target gene interaction networks were constructed using the Cytoscape software (3.6.1 version),^[23]^ with the top 5 up- and down-regulated circRNAs and their putative target miRNAs and mRNAs. The miRNAs were predicted using CircInteractome, and the 5 highest scoring (highest context + score percentile) miRNAs were included in the network. In addition, the co-expressing miRNAs extracted from the literature search were also included. The mRNAs targeted by the miRNAs were predicted using miRanda (http://www.microrna.org/) and the top 5 mRNAs (with the lowest mirSVR score) were selected, along with those obtained from literature search. To further validate the analysis, some target mRNAs were verified using the Oncomine platform.

### 2.3 Gene ontology (GO) and Kyoto Encyclopedia of Genes and Genomes (KEGG) pathway analyses

The Database for Annotation, Visualization, and Integrated Discovery (https://david.ncifcrf.gov/, version 6.8) online tool^[24,25]^ was used to identify the origin genes of the DECRs, and their putative functions were determined using the gene ontology (GO) and Kyoto Encyclopedia of Genes and Genomes (KEGG) analyses. The gene functions were determined on the basis of the 3 GO terms – biological process, cellular component (CC), and molecular function. The associated pathways were elucidated using KEGG analysis. *P* < .05 indicated the significantly enriched GO terms and KEGG pathways.

### 2.4 Samples collection

A total of 22 CSCC and 9 normal cervical tissue samples were collected from August 2018 to September 2019 at the First Affiliated Hospital of Guangxi Medical University for quantitative real-time PCR (qRT-PCR). The CSCC patients were in stages IB1–IIA1, did not present any other malignancies, and had not received chemotherapy or radiotherapy before samples collection. The diagnosis of CSCC was affirmed by a pathologist, and written informed consent was obtained from all patients. The study was approved by the Ethics Committee of the first affiliated Hospital of Guangxi Medical University.

### 2.5 RNA isolation and reverse transcription

Total RNA was extracted from the tissues using the TRIzolReagen (TAKARA) according to the manufacturer's instructions, and quantified using NanoDropND-2000 Lite spectrophotometer (Thermo Scientific). The purity and concentration of each RNA sample were in the normal range. CircRNA cDNA was synthesized using the PrimeScript RT Reagent Kit with gDNA Eraser (Takara, Dalian, Liaoning, China).

### 2.6 Quantitative real-time PCR (qRT-PCR)

QRT-PCR was performed using the TB Green Premix Ex Taq II (TaKaRa) on the Applied Biosystems7500 Fast Real-Time PCR System (Thermo Fisher Scientific). The reaction conditions were 95 °C for 30 seconds, and 40 cycles at 95°C for 5 seconds and 60°C for 30 seconds. Melting curves were plotted and the relative expression levels were calculated using the 2^−ΔΔCT^ method with 18S rRNA as the endogenous control. The primers were synthesized by Takara biotechnology (Dalian, China) and the sequences are listed in Table 1.

**Table 1:** Primer sequences of the circRNAs.

Gene	Forward primer (5′–3′)	Reverse primer (5′–3′)
hsa_circ_0000745	ATGTTGAAAGTAGCCCGAGCAG	TGGGAGTGTTGGAAGAAGTTGG
hsa_circ_0084927	CAAAGCAACAGGTGAAGATTTCC	GTAAACCTCGTGCCCTGACTAC
hsa_circ_0002762	ATGCCCAGAAATGGAAGCA	TCCTCGGAGTGTGAGGGATAG
hsa_circ_0075341	GCCTTCAGCACAGATGCAG	TGAAGGTTGAGTCTGCCACTT
hsa_circ_0007905	GGCTGGACAATGTGATGAAGAA	AGCTAATGCCTGCACAGATGAA
hsa_circ_0031027	ACCTCCATCGAACCCATCC	CCCCACAGCAAGCCAAA
hsa_circ_0065898	GTCTGCACTTTGGGGAATGAA	CACAGCAGGTGAAGCCACTC
hsa_circ_0070190	CTTCCTTTTGGTGAGAGTCAGGTT	ACATTGAGGTCTGGCATGTTATTG
hsa_circ_0078383	GTGCTTGTTAAATGTGCTGTGTTG	ACAGAGGAATGTGGAGGACACTT
18S	CCAGTAAGTGCGGGTCATAAG	GGCCTCACTAAACCATCCAA

circRNAs = circular RNAs.

### 2.7 Statistical analysis

Statistical analysis was performed using SPSS 20.0 software (IBM, Chicago, IL). Independent *t* test or Chi-square test was used to compare different groups. *P* value < .05 was considered statistically significant.

## 3 Results

### 3.1 CSCC is associated with an altered circRNA profile

**Figure 1:**
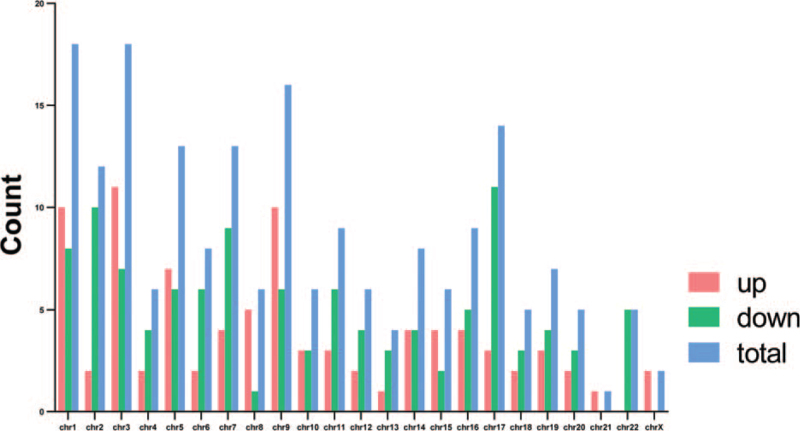
The chromosomal distribution of DECRs in CSCC. CSCC = cervical squamous cell carcinoma, DECRs = differentially expressed circRNAs.

**Figure 2:**
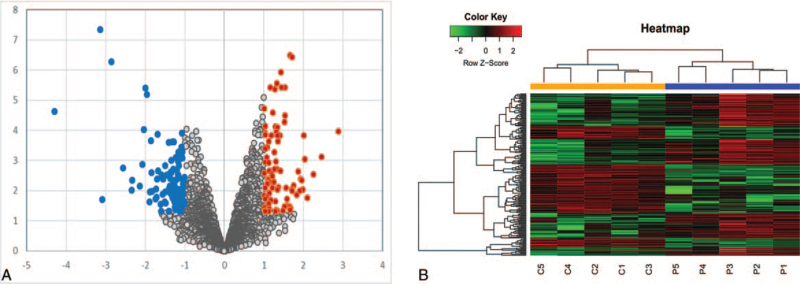
(A) Volcano plots showing the DECRs. Gray dots indicate circRNAs with no significant difference, red dots indicate significant up-regulated circRNAs, and blue dots indicated significant down-regulated circRNAs. (B) Heat map showing DECR profile. Each row represents 1 circRNA, and each column represents 1 sample. Red color indicates up-regulation and green indicates down-regulation. circRNAs = circular RNAs, DECRs = differentially expressed circRNAs.

We identified a total of 197 DECRs in the CSCC tissues relative to normal cervix tissues, of which 87 were up-regulated and 110 down-regulated. The DECRs were validated on the basis of their sequences using CircBase (http://www.circbase.org/). The top 40 DECRs are listed in Table 2, and their chromosomal distribution is illustrated in Figure 1. To further determine the trend of these DECRs in CSCC, we performed hierarchical clustering analysis using R package “gplot”, and the volcano plot is shown in Figure 2. We predicted 291 and 323 target miRNAs of the up- and downregulated circRNAs respectively, along with an additional 205 miRNAs correlated to CC through a literature search. Venn analysis identified 37 co-expressing miRNAs for the up-regulated circRNAs and 39 for the down-regulated circRNAs. At least 5 of the most likely target mRNAs for each miRNA were selected for ceRNA network analysis.

**Table 2:** Top 40 differently expressed circRNAs in cervical squamous cell carcinoma.

circRNA	*P* values	logFC	Regulation	Chromosome	Hostgene
hsa_circ_0000745	1.07 E-04	2.89	Up	chr17	SPECC1
hsa_circ_0084927	7.47 E-04	2.46	Up	chr8	ESRP1
hsa_circ_0002762	2.89 E-03	2.26	Up	chr12	CDK17
hsa_circ_0075341	1.73 E-02	2.1	Up	chr5	MAPK9
hsa_circ_0081672	9.17 E-04	2.03	Up	chr7	POLR2J
hsa_circ_0084904	1.50 E-04	2.01	Up	chr8	KIAA1429
hsa_circ_0001461	9.19 E-03	1.98	Up	chr4	FAT1
hsa_circ_0085616	4.53 E-03	1.96	Up	chr8	ASAP1
hsa_circ_0003037	4.15 E-03	1.94	Up	chr5	TRIO
hsa_circ_0085923	1.01 E-02	1.91	Up	chr8	PLEC
hsa_circ_0001849	2.27 E-03	1.87	Up	chr9	UBAP2
hsa_circ_0020460	1.36 E-02	1.83	Up	chr10	DOCK1
hsa_circ_0007905	6.67 E-03	1.76	Up	chr1	STX6
hsa_circ_0008285	3.74 E-07	1.72	Up	chr6	CDYL
hsa_circ_0008812	8.89 E-03	1.72	Up	chr9	RAD23B
hsa_circ_0005360	1.01 E-02	1.72	Up	chr19	LDLR
hsa_circ_0001776	3.91 E-02	1.68	Up	chr7	ESYT2
hsa_circ_0067717	4.43 E-02	1.67	Up	chr3	RNF13
hsa_circ_0000520	3.29 E-07	1.66	Up	chr14	RPPH1
hsa_circ_0002151	3.37 E-02	1.65	Up	chr15	PDIA3
hsa_circ_0031027	2.39 E-05	−4.29	Down	chr13	TMCO3
hsa_circ_0065898	4.51 E-08	−3.14	Down	chr3	VPRBP
hsa_circ_0046290	1.98 E-02	−3.09	Down	chr17	ASPSCR1
hsa_circ_0070190	5.20 E-07	−2.86	Down	chr4	BMP2K
hsa_circ_0027821	1.77E-03	−2.56	Down	chr12	RMST
hsa_circ_0078383	9.65 E-03	−2.36	Down	chr6	TIAM2
hsa_circ_0058794	4.48 E-03	−2.33	Down	chr2	AGAP1
hsa_circ_0077248	7.11 E-03	−2.14	Down	chr6	SNX14
hsa_circ_0000301	1.32 E-03	−2.07	Down	chr11	SPI1
hsa_circ_0043280	9.63 E-05	−2.03	Down	chr17	TADA2A
hsa_circ_0042986	4.07 E-06	−2	Down	chr17	SUZ12
hsa_circ_0000077	6.52 E-06	−1.96	Down	chr1	TM2D1
hsa_circ_0004547	2.37 E-02	−1.89	Down	chr22	KREMEN1
hsa_circ_0064735	1.08 E-02	−1.88	Down	chr3	UBP1
hsa_circ_0020926	2.23 E-04	−1.85	Down	chr11	STIM1
hsa_circ_0062432	2.46 E-03	−1.84	Down	chr22	YPEL1
hsa_circ_0038645	1.06 E-02	−1.84	Down	chr16	PRKCB
hsa_circ_0032641	1.91 E-02	−1.74	Down	chr14	MLH3
hsa_circ_0003503	8.93 E-03	−1.73	Down	chr5	ADAMTS6
hsa_circ_0031419	1.68 E-02	−1.71	Down	chr14	SCFD1

circRNAs = circular RNAs, FC = fold change.

### 3.2 Functional analysis of DECRs

**Figure 3:**
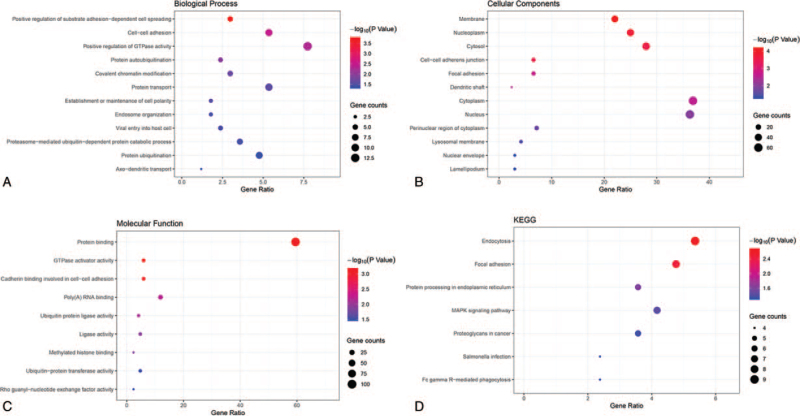
Functional annotation of the parent genes of DECRs in CSCC. The bubble chart shows GO and KEGG enrichment terms (*P* value <.05). (A) BP, (B) CC (C) MF, (D) KEGG. BP = biological proves, CC = cellular component, CSCC = cervical squamous cell carcinoma, DECRs = differentially expressed circular RNAs, GO = gene ontology, KEGG = Kyoto Encyclopedia of Genes and Genomes.

**Figure 4:**
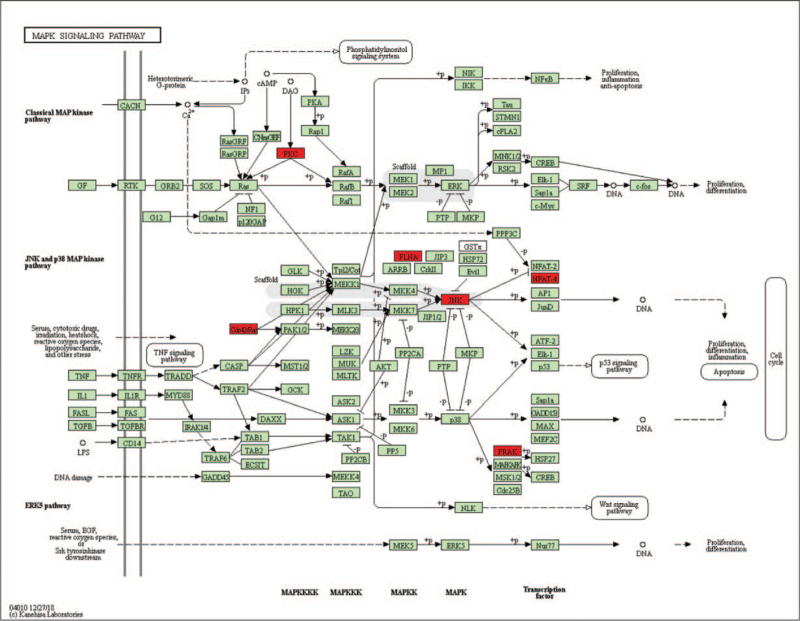
MAPK signaling pathway is the significant pathway of the source genes (red background) of DECRs in CSCC. CSCC = cervical squamous cell carcinoma, DECRs = differentially expressed circular RNAs.

**Figure 5:**
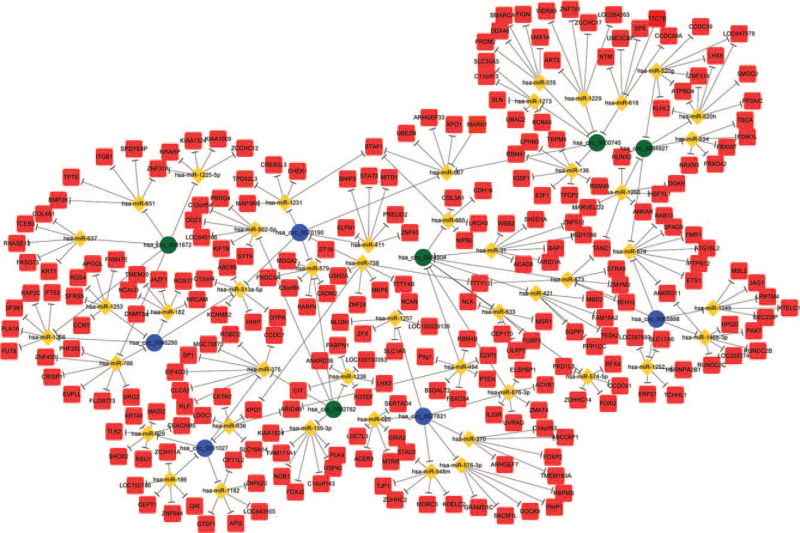
The ceRNA network of 5 most up-regulated and down-regulated circRNAs. The network consists of 10 circRNAs, 52 miRNAs and 311 mRNAs. The circRNAs are marked by circle, rhombi represent miRNA, and squares represent mRNAs. Cyan represents up- regulated circRNAs, and blue represents down-regulated circRNAs. Solid lines represent relationship between 2 nodes. circRNAs = circular RNAs.

The DECRs are derived from 182 genes, and their functional analysis revealed the following most significantly enriched GO terms: molecular function – protein binding, GTPase activator activity and cadherin binding involved in cell–cell adhesion, biological process – positive regulation of substrate adhesion-dependent cell spreading, cell–cell adhesion and positive regulation of GTPase activity, and CC – membrane, nucleoplasm, and cytosol (Fig. 3). The KEGG pathway analysis indicated that endocytosis, focal adhesion, and protein processing in endoplasmic reticulum, and the MAPK signaling pathway were the most enriched. The latter is significantly correlated with CSCC, and includes the CDC42, MAPKAPK5, MAPK9, FLNB, NFATC3, FLNA, and PRKCB genes (Fig. 4). We next constructed a circRNA–miRNA–mRNA interaction network to better elucidate the function of the DECRs in CSCC. The ceRNA network of the 5 most significant up-and down-regulated circRNAs is shown in Figure 5.

### 3.3 Validation of DECRs

**Figure 6:**
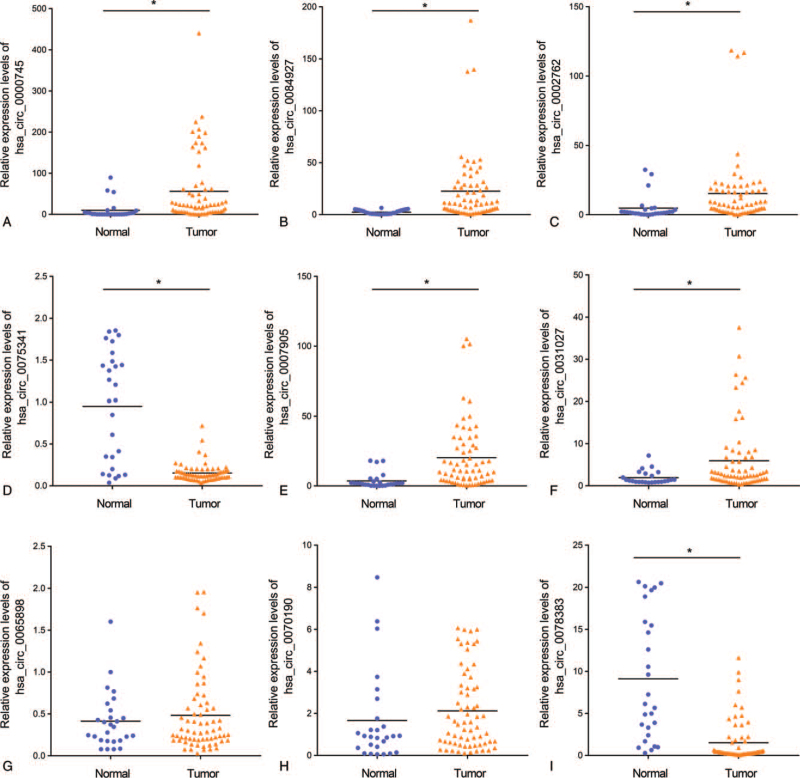
qRT-PCR analysis of the candidate circRNAs in the CC patients and normal controls. Hsa_circ_0000745 (A), hsa_circ_0084927 (B), hsa_circ_0002762 (C), hsa_circ_0007905 (E) and hsa_circ_0031027 (F) were upregulated in the CSCC patients compared with the normal controls. Hsa_circ_0075341 (D) and hsa_circ_0078383 (I) were downregulated in the CSCC patients. Hsa_circ_0065898 (G) and hsa_circ_0070190 (H) were similar in both groups. ^∗^*P* < .05. CC = cellular component, circRNAs = circular RNAs, CSCC = cervical squamous cell carcinoma, qRT-PCR = quantitative real-time PCR.

To verify the results of circRNAs sequencing and determine the possible mechanisms of circRNAs in the development of the occurrence of CSCC, the expression levels of 5 up-regulated and 4 down-regulated DECRs were verified. A total of 22 CSCC and 9 normal cervical tissue samples were used for validation, and each sample was repeated 3 times PCR. Hsa_circ_0000745, hsa_circ_0084927, hsa_circ_0002762, hsa_circ_0007905 were highly upregulated (Fig. 6A–C, E) and hsa_circ_0078383 was downregulated (*P* < .05, Fig. 2I) in CSCC tissues, which was consistent with the high-throughput sequencing results. In contrast, the expression levels of hsa_circ_0065898, and hsa_circ_0070190 were similar between the CSCC and normal tissues (*P* = .48, Fig. 6G; *P* = .31, Fig. 6H). In addition, hsa_circ_0075341 (*P* < .05, Fig. 6D) and hsa_circ_0031027, (*P* < .05, Fig. 6F) were inversely expressed in CSCC and normal tissues.

## 4 Discussion

The rapid development of bioinformatics and high-throughput sequencing in recent years has helped identify several circRNAs, which regulate gene expression by acting as ceRNAs that “absorb” target miRNAs.^[12]^ Studies have shown a clear role of miRNAs in CC progression and prognosis. For instance, Zhou et al^[26]^ revealed that miRNA-218 inhibited CC by blocking immune escape of the tumor cellsvia IDO1 downregulation. Persistent infection of the cervical squamous or glandular epithelium by oncogenic human papillomaviruses is a major risk factor of CC. Zheng et al^[27]^ showed that HPV16 E7 regulated the expression profiles of circRNAs in CC cells. Although circRNAs are increasingly being considered as biomarkers for tumor diagnosis and targeted therapy,^[28,29]^ not much is known regarding their role in CC. To this end, we identified 197 DECRs between CSCC and para-cancer tissue with GEO datasets, and further validated the overexpression of hsa_circ_0000745, hsa_circ_0084927, hsa_circ_0002762, and hsa_circ_0007905, and the downregulation of hsa_circ_0078383 in CSCC tissues relative to normal tissues. In contrast, the expression of hsa_circ_0065898, hsa_circ_0070190, hsa_circ_0075341, and hsa_circ_0031027 in CSCC and normal cervical tissues need further verification with a larger sample size. At the same time, the errors of high-throughput sequencing cannot be ruled out.

Eighty-seven circRNAs were significantly upregulated in CSCC, including hsa_circ_0000745, has_circ_0023404, and hsa_circ_0084927. Hsa_circ_0000745 was targeted by hsa-miR-136, hsa-miR-1229, hsa-miR-1273, and hsa-miR-618. Through Oncomine analysis, we found that the target genes of hsa-miR-136 included E2F1, TFCP2 and RBM41, all of which are over-expressed in CSCC. Lu et al^[30]^ reported that miR-136 inhibited proliferation and promoted apoptosis and radio-sensitivity in cervical carcinoma cells by targeting E2F1 through the NF-κB signaling pathway, and was associated with improved prognosis. Recently, Jiao et al^[31]^ discovered that hsa_circ_0000745 promotes the development of CC. In addition, hsa_circ_0023404 inhibits proliferation, cell cycle progression, migration, and invasion of CC cells via the mir-136/TFCP2/YAP pathway.^[17]^ Hsa_circ_0084927 was targeted by hsa-miR-520h, hsa-miR-874, hsa-miR-634, hsa-miR-1250, and hsa-miR-520g. Studies have correlated mir-520h to the occurrence, progression, invasion, and metastasis of ovarian, breast, lung, and pancreatic cancers.^[32–35]^ The target genes of hsa-miR-520h were KLHL2, ATPBD4, LHX8, and ZNF318, which are also expressed at high levels in CSCC. Chang et al^[36]^ showed that overexpression of miR-520h is related to CC metastasis. Taken together, overexpression of hsa_circ_0000745, hsa_circ_0023404, and hsa_circ_0084927 promotes CSCC cell differentiation, proliferation, and metastasis.

Hsa_circ_0078383 was targeted by hsa-miR-520h, hsa-miR-1197, hsa-miR-1298, and hsa-miR 520g. The target mRNAs of hsa-miR-520h included PP2A/C, KLHL2, ATPBD4, LOC647979, LHX8, ZNF318, and SMOC2, of which PP2A, KLHL2, ATPBD4, ZNF318, and SMOC2 are significantly downregulated in CC by verified in Oncomine platform. Chang et al.^[36]^ showed that miR-520h promotes metastasis of CC cells by downregulating PP2A. Hsa_circ_0065898 was significantly downregulated in the CSCC tissues, and was targeted by hsa-miR-1245, hsa-miR-1252, hsa-miR-146b-3p, hsa-miR-873, and hsa-miR-874. The target mRNAs of hsa-miR-146b-3p included RUNDC2B, RUNDC2C, PAK7, ANKRD11, LOC203274, and HPGD, all of which except LOC203274, are significantly downregulated in CC. Yao et al^[37]^ reported that miR-146b-3p promotes the development of CC by downregulating HPGD.

The parent genes of the DECRs were significantly enriched for functions like positive regulation of substrate adhesion-dependent cell spreading, metabolism, positive regulation of GTPase activity, protein regulation, and intercellular adhesion, all of which are related to the proliferation and invasion of tumor cells. KEGG pathway analysis showed significant enrichment of the mitogen-activated protein kinases (MAPK) signaling pathway. Dysregulated MAPK signaling pathway is associated with cancer-related functions like tumor cell proliferation, differentiation, migration, senescence, and apoptosis.^[38]^ PRKCB, the parent gene of hsa_circ_0038645, regulates the MAPK signaling pathway by phosphorylating the RAS signaling pathway (Fig. 4). The expression of PRKCB was related to the survival of CC patients in TGCA database (*P* = .004). Since both pathways influence and regulate each other, we surmise that the Ras-MAPK axis plays a key role in CSCC progress and metastasis.

## 5 Conclusions

We identified 197 DECRs in CSCC, and established the circRNA-associated ceRNA network that can help unearth novel diagnostic and therapeutic biomarkers, and elucidate the mechanistic basis of CSCC.

## Acknowledgments

Authors thank the women participated in our study.

## Author contributions

All authors substantially contributed to the manuscript. JH: protocol/project development, data collection and management, sample collection, experiment operation, data analysis, and manuscript writing/editing (original draft). JC: protocol/project development, conceptualization, funding acquisition, investigation, methodology, analysis, project administration, supervision, validation, and writing (review/edits). QH: sample collection, performed the analysis writing (review/edits).

**Writing – original draft:** Jinbing Huang, Junying Chen, Qiaoqiao Huang.

## References

[1] Ferlay J Colombet M Soerjomataram I. Estimating the global cancer incidence and mortality in 2018: GLOBOCAN sources and methods. Int J Cancer 2019;144:1941–53. 30350310 10.1002/ijc.31937

[2] Tewari KS Sill MW Long HJ III. Improved survival with bevacizumab in advanced cervical cancer. N Engl J Med 2014;370:734–43. 24552320 10.1056/NEJMoa1309748 PMC4010094

[3] Ferlay J Steliarova-Foucher E Lortet-Tieulent J. Cancer incidence and mortality patterns in Europe: estimates for 40 countries in 2012. Eur J Cancer 2013;49:1374–403. 23485231 10.1016/j.ejca.2012.12.027

[4] Li H Wu X Cheng X. Advances in diagnosis and treatment of metastatic cervical cancer. J Gynecol Oncol 2016;27:e43. 27171673 10.3802/jgo.2016.27.e43 PMC4864519

[5] Basu P Mehta A Jain M. A randomized phase 2 study of ADXS11-001 listeria monocytogenes–listeriolysin O immunotherapy with or without cisplatin in treatment of advanced cervical cancer. Int J Gynecol Cancer 2018;28:764–72. 29538258 10.1097/IGC.0000000000001235 PMC5929492

[6] Luo C Liu M Li X. Efficacy and safety outcomes of robotic radical hysterectomy in Chinese older women with cervical cancer compared with laparoscopic radical hysterectomy. BMC Women Health 2018;18:01–5. 10.1186/s12905-018-0544-x PMC5930733 29716555

[7] Obrzut B Kusy M Semczuk A Obrzut M Kluska J. Prediction of 5–year overall survival in cervical cancer patients treated with radical hysterectomy using computational intelligence methods. BMC Cancer 2017;17:01–9. 10.1186/s12885-017-3806-3 PMC5727988 29233120

[8] Marquina G Manzano A Casado A. Targeted agents in cervical cancer: beyond bevacizumab. Curr Oncol Rep 2018;20:01–10. 10.1007/s11912-018-0680-3 29611060

[9] Memczak S Jens M Elefsinioti A. Circular RNAs are a large class of animal RNAs with regulatory potency. Nature 2013;495:333–8. 23446348 10.1038/nature11928

[10] Hentze MW Preiss T. Circular RNAs: splicing's enigma variations. EMBO J 2013;32:923–5. 23463100 10.1038/emboj.2013.53 PMC3616293

[11] Barrett SP Salzman J. Circular RNAs: analysis, expression and potential functions. Development 2016;143:1838–47. 27246710 10.1242/dev.128074 PMC4920157

[12] Hansen TB Jensen TI Clausen BH. Natural RNA circles function as efficient microRNA sponges. Nature 2013;495:384–8. 23446346 10.1038/nature11993

[13] Xie F Li Y Wang M. Circular RNA BCRC-3 suppresses bladder cancer proliferation through miR-182-5p/p27 axis. Mol Cancer 2018;17:01–12. 10.1186/s12943-018-0892-z PMC6169039 30285878

[14] Zhou X Natino D Qin Z. Identification and functional characterization of circRNA-0008717 as an oncogene in osteosarcoma through sponging miR-203. Oncotarget 2018;9:22288. 29854278 10.18632/oncotarget.23466 PMC5976464

[15] Tian X Zhang L Jiao Y Chen J Shan Y Yang W. CircABCB10 promotes nonsmall cell lung cancer cell proliferation and migration by regulating the miR-1252/FOXR2 axis. J Cell Biochem 2019;120:3765–72. 30417418 10.1002/jcb.27657 PMC6587869

[16] Li M Wang Y Liu Y Zhang X Liu J Wang P. Low expression of hsa_circ_0018069 in human bladder cancer and its clinical significance. Biomed Res Int 2019;2019:9681863. 30984788 10.1155/2019/9681863 PMC6431508

[17] Zhang J Zhao X Zhang J Zheng X Li F. Circular RNA hsa_circ_0023404 exerts an oncogenic role in cervical cancer through regulating miR-136/TFCP2/YAP pathway. Biochem Biophys Res Commun 2018;501:428–33. 29738762 10.1016/j.bbrc.2018.05.006

[18] Liu J Wang D Long Z Liu J Li W. CircRNA8924 promotes cervical cancer cell proliferation, migration and invasion by competitively binding to MiR-518d-5p/519-5p family and modulating the expression of CBX8. Cell Physiol Biochem 2018;48:173–84. 30007986 10.1159/000491716

[19] Li S Teng S Xu J. Microarray is an efficient tool for circRNA profiling. Brief Bioinform 2019;20:1420–33. 29415187 10.1093/bib/bby006

[20] Edgar R Domrachev M Lash AE. Gene Expression Omnibus: NCBI gene expression and hybridization array data repository. Nucleic Acids Res 2002;30:207–10. 11752295 10.1093/nar/30.1.207 PMC99122

[21] Barrett T Wilhite SE Ledoux P. NCBI GEO: archive for functional genomics data sets—update. Nucleic Acids Res 2012;41:D991–5. 23193258 10.1093/nar/gks1193 PMC3531084

[22] Dudekula DB Panda AC Grammatikakis I De S Abdelmohsen K Gorospe M. CircInteractome: a web tool for exploring circular RNAs and their interacting proteins and microRNAs. RNA Biol 2016;13:34–42. 26669964 10.1080/15476286.2015.1128065 PMC4829301

[23] Shannon P Markiel A Ozier O. Cytoscape: a software environment for integrated models of biomolecular interaction networks. Genome Res 2003;13:2498–504. 14597658 10.1101/gr.1239303 PMC403769

[24] Sherman BT Lempicki RA. Systematic and integrative analysis of large gene lists using DAVID bioinformatics resources. Nat Protoc 2009;4:44. 19131956 10.1038/nprot.2008.211

[25] Huang DW Sherman BT Lempicki RA. Bioinformatics enrichment tools: paths toward the comprehensive functional analysis of large gene lists. Nucleic Acids Res 2009;37:01–13. 10.1093/nar/gkn923 PMC2615629 19033363

[26] Zhu L Tu H Liang Y Tang D. MiR-218 produces anti-tumor effects on cervical cancer cells in vitro. World J Surg Oncol 2018;16:01–10. 10.1186/s12957-018-1506-3 PMC6186038 30314496

[27] Zheng S-R Zhang H-R Zhang Z-F. Human papillomavirus 16 E7 oncoprotein alters the expression profiles of circular RNAs in Caski cells. J Cancer 2018;9:3755. 30405847 10.7150/jca.24253 PMC6216014

[28] Xu L Zhang M Zheng X Yi P Lan C Xu M. The circular RNA ciRS-7 (Cdr1as) acts as a risk factor of hepatic microvascular invasion in hepatocellular carcinoma. J Cancer Res Clin Oncol 2017;143:17–27. 27614453 10.1007/s00432-016-2256-7 PMC11819007

[29] Li P Chen S Chen H. Using circular RNA as a novel type of biomarker in the screening of gastric cancer. Clin Chim Acta 2015;444:132–6. 25689795 10.1016/j.cca.2015.02.018

[30] Lu H-J Jin P-Y Tang Y. microRNA-136 inhibits proliferation and promotes apoptosis and radiosensitivity of cervical carcinoma through the NF-κB pathway by targeting E2F1. Life Sci 2018;199:167–78. 29452167 10.1016/j.lfs.2018.02.016

[31] Jiao J Zhang T Jiao X. hsa_circ_0000745 promotes cervical cancer by increasing cell proliferation, migration, and invasion. J Cell Physiol 2020;235:1287–95. 31256433 10.1002/jcp.29045 PMC6899562

[32] Zhang J Liu W Shen F. The activation of microRNA-520h–associated TGF-β1/c-Myb/Smad7 axis promotes epithelial ovarian cancer progression. Cell Death Dis 2018;9:01–15. 10.1038/s41419-018-0946-6 PMC6115398 30158641

[33] Su C-M Wang M Hong C. miR-520h is crucial for DAPK2 regulation and breast cancer progression. Oncogene 2016;35:1134–42. 25982274 10.1038/onc.2015.168

[34] Yu Y Chen H Chen P. MiR-520h-mediated FOXC2 regulation is critical for inhibition of lung cancer progression by resveratrol. Oncogene 2013;32:431–43. 22410781 10.1038/onc.2012.74

[35] Wang F Xue X Wei J. hsa-miR-520h downregulates ABCG2 in pancreatic cancer cells to inhibit migration, invasion, and side populations. Br J Cancer 2010;103:567–74. 20628378 10.1038/sj.bjc.6605724 PMC2939772

[36] Chang Y-W Chen M-W Chiu C-F. Arsenic trioxide inhibits CXCR4-mediated metastasis by interfering miR-520h/PP2A/NF-κB signaling in cervical cancer. Ann Surg Oncol 2014;21:687–95. 10.1245/s10434-014-3812-5 25047463

[37] Yao S Xu J Zhao K. Down-regulation of HPGD by miR-146b-3p promotes cervical cancer cell proliferation, migration and anchorage-independent growth through activation of STAT3 and AKT pathways. Cell Death Dis 2018;9:01–10. 10.1038/s41419-018-1059-y PMC6192999 30333561

[38] Sun Y Liu W-Z Liu T Feng X Yang N Zhou H-F. Signaling pathway of MAPK/ERK in cell proliferation, differentiation, migration, senescence and apoptosis. J Recept Signal Transduct Res 2015;35:600–4. 26096166 10.3109/10799893.2015.1030412

